# *Pediculus humanus capitis*: a study of the male genitalia using a combined stereoscopic, confocal laser scanning, and scanning electron microscopy approach

**DOI:** 10.1186/s13071-021-05082-w

**Published:** 2021-11-17

**Authors:** Blanca E. Álvarez-Fernández, María Morales-Suárez-Varela, Benjamín Nogueda-Torres, M. Adela Valero

**Affiliations:** 1 Departamento de Parasitología, Facultad de Farmacia, Universitat de Valencia, Avenida Vicente Andrés Estellés s/n, 46100 Burjassot, Valencia, Spain; 2 Facultad de Ciencias Químico Biológicas, Universidad Autónoma de Guerrero, Avenida Lázaro Cárdenas S/N, Ciudad Universitaria, 39090 Chilpancingo, Guerrero Mexico; 3 Departamento de Medicina Preventiva y Salud Pública, Ciencias de la Alimentación, Toxicología y Medicina Legal, Facultad de Farmacia, Universitat de Valencia, Avenida Vicente Andrés Estellés s/n, 46100 Burjassot, Valencia, Spain; 4 Consorcio Para la Investigación Biomédica en Red de Epidemiología y Salud Pública (CIBER Epidemiología y Salud Pública-CIBERESP), Avenida Monforte de Lemos 3-5, Pabellón 11, Planta 0, 28029 Madrid, Spain; 5 Departamento de Parasitología, Escuela Nacional de Ciencias Biológicas, Instituto Politécnico Nacional, Prolongación de Carpio y Plan de Ayala S/N, Miguel Hidalgo, Santo Tomás, 11340 Mexico City, Mexico

**Keywords:** Copulation, Genital chamber, Dilator, Genitalia, *Pediculus humanus capitis*, Vesica, Confocal laser scanning microscopy, Scanning electron microscopy

## Abstract

**Background:**

The male genital structures of arthropods are key features in the taxonomic and phylogenetic study of these organisms. The male genitalia of the head louse *Pediculus humanus capitis* are complex organs which are partly composed of structures that dynamically extrude during copulation.

**Methods:**

Here, we describe the morphology of the genitalia of *P. humanus capitis* at the copulation stage, and at rest, by using stereoscopic microscopy, confocal laser scanning microscopy (CLSM), and scanning electron microscopy (SEM).

**Results:**

CLSM and SEM images revealed that the vesica is composed of two distinct anatomical parts, the proximal lobe and the distal lobe. Both lobes have short and narrow spines, as well as long and wide scales with either sharp or rounded tips. The rounded scales vary in size and have a wavy base and rounded tips, and thus resemble a tongue in appearance. We identified a gland-like area on the penis with 11 shallow circular depressions, and a flat area with 14–16 exit orifices. The apical end of the penis has a foliaceous trifurcation and serves to expel the contents of the ejaculatory duct. These characteristics were recorded for all the specimens analyzed, indicating that these structures are highly conserved; to our knowledge, they have not been previously reported for any suborder of lice.

**Conclusions:**

To the best of our knowledge, our results reveal for the first time the morphological details, and complexity, of the male genitalia of the head louse *P. humanus capitis* at different stages of copulation. The new approach described here provided information that should be taken into consideration in future research on the genitalia of lice. Application of this approach will also impact the taxonomic and phylogenetic study of other insect taxa.

**Graphical Abstract:**

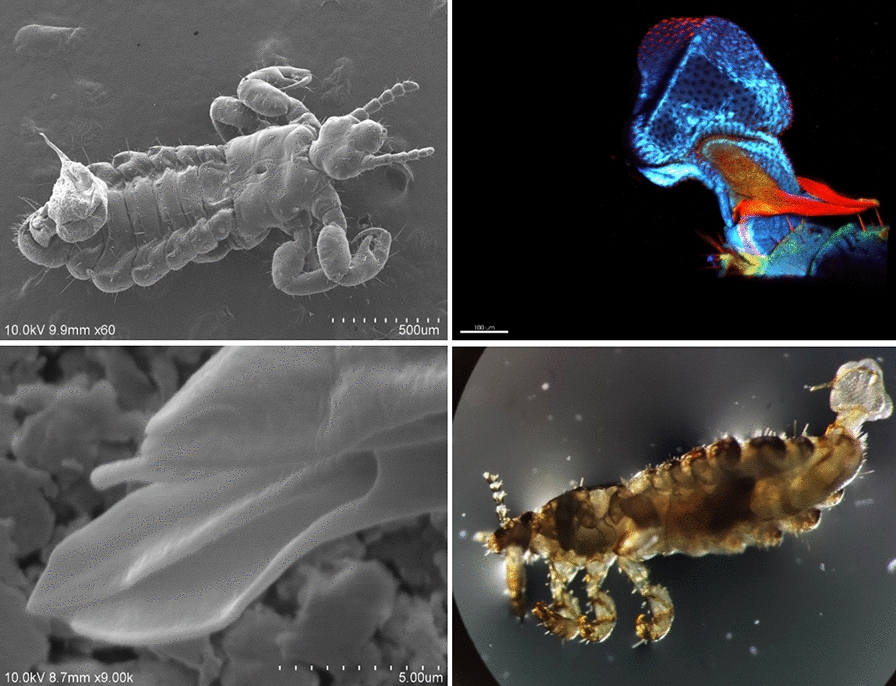

## Background

Lice are ectoparasites of birds and mammals which spend their entire life cycle on the host’s body [1]. Lice of the family Pediculidae, suborder Anoplura, include two ecotypes that exclusively infest humans [2–4]: *Pediculus humanus capitis*, described by De Geer in 1778; and *Pediculus humanus humanus*, described by Linnaeus in 1758. *Pediculus humanus capitis* is a cosmopolitan ectoparasite that causes pediculosis, one of the most common diseases caused by arthropods [5]. *Pediculus humanus capitis* inhabits the scalp, while *P. humanus humanus* is limited to the body; even though these species of lice are ecologically different, they are morphologically and biologically similar [6].

Sexual reproduction plays a major role in the evolution of body structures, sexual relationships, and behavior [7]. In beetles, the study of male (and, less frequently, female) genitalia has often been included in taxonomic and phylogenetic research [8]. In the Pediculidae, the male genitalia have been the target structures of multiple studies, with the genitalia of the Anoplura being used as one of the most common research models [9]. The male phallic organ of lice is a highly complex structure; it is stored in the genital chamber and, therefore, is not exposed to the external environment [1]. This hinders detailed study of the microstructure of the male genitalia.

In the seminal work of Nutall, published in 1917 [10], the copulatory apparatus of the male and the copulation process were described for living specimens of body lice. Visual representations of other anatomical parts were also published [10]. According to Nutall [10], the principal structures of the extruded genital apparatus are the dilator, the vesica of the penis, the support of the penis, the penis, and the ejaculatory duct. In 1983, Hatsushika et al. [11] investigated the external anatomy of male and female *P. humanus capitis* by scanning electron microscopy (SEM). In that work, a sclerotic structure was documented, which the authors called a pseudopenis (and Nutall called the dilator), without delving into its description [11].

However, there is no standardized nomenclature for the structures of the male genitalia of the order Phthiraptera [9]. Furthermore, structures of great morphological significance, as described by Nutall in 1917 [10], are either no longer mentioned in studies or there is no conclusive evidence that they have been studied [12].

For many species of sucking lice, the structure, and relationships, of the finer parts of the genitalia have not been described, and homologies have not been defined or only very weakly established [12]. Currently, the information available on the male genitalia of *P. humanus capitis* is limited to that on a few structures such as the basal plate and the main sclerites. The shape of the subgenital plate is of taxonomic importance at the species level, while the shape, length, and thickness of the parameres play important roles at both the genus and species level [13]. The limited morphological descriptions that have so far been obtained through the use of modern microscopic approaches and techniques hinder the integration of knowledge on the male genitalia of lice. The use of state-of-the-art techniques can positively impact the taxonomic and phylogenetic research on insects.

The objective of this work is to investigate and describe the morphology of the structures that form the male genitalia of *P. humanus capitis* both at rest and at copulation. We also want to compare and confirm the terminology and morphology described here with that from other investigations on lice. For this, a combination of stereoscopic microscopy (SM), confocal laser scanning microscopy (CLSM) and SEM was applied, to our knowledge for the first time, to the study of the male genitalia of lice.

## Methods

### Specimens

Adult *P. humanus capitis* lice (*n* = 506) obtained from 50 girls aged 7–14 years were studied. The collection was carried out from June 2019 to December 2019 in the city of Chilpancingo, Guerrero, Mexico. The specimens were collected at different times of the day by dry combing with a fine-toothed metal comb. The specimens were kept in Eppendorf tubes filled with Karnovsky fixative (25% paraformaldehyde and 0.5% glutaraldehyde). The specimens were separated into males and females under a stereoscopic microscope.

Morphological identification was conducted in the Parasitology Laboratory of the Department of Parasitology of the University of Valencia, Spain. For this study, 197 males were analyzed. Of the total samples of male lice, 187 presented genitalia at rest, while 10 had extruded genitalia at different stages. A stereomicroscope (Nikon SMZ-U; Nikon Instruments, Tokyo, Japan), a confocal laser scanning microscope (Olympus FV1000-IX81; Olympus, Tokyo), and a scanning electron microscope (Hitachi S4800; Hitachi High-Technologies, Tokyo) were used for the morphological characterization of the specimens. The samples were prepared according to the methodology of the Central Service of Support for Experimental Research of the University of Valencia, Spain.

### Stereoscopic microscopy

First, the 197 specimens were individually mounted on Petri dishes with Karnovsky fixative to prevent dehydration. To visualize the specimens, ×0.75 and ×7.5 objectives were used, with the focus and illumination adjusted for each sample. Genitalia at rest were identified in 187 of the specimens. Extruded genitalia at different stages (two at pre-copulation and eight at copulation stages) were identified for 10 specimens. The entire body and the genitals were photographed. All of the samples were stored in Eppendorf tubes with fresh Karnovsky fixative. Subsequently, the 10 males with extruded genitalia were prepared for the two other microscopic visualization methods, CLSM and SEM.

### Confocal laser scanning microscopy

The 10 male samples were individually mounted on Petri dishes with Karnovsky fixative to avoid dehydration. An Olympus FV1000-IX81 confocal laser scanning microscope (Olympus) was used, with a 10 × 2 objective lens. Using the autofluorescence of the samples, the areas of interest were scanned per individual. Wavelengths corresponding to the blue (excitation = 405 nm and emission = 425–475 nm), green (excitation = 488 nm and emission = 500–545 nm), and red channels (excitation = 559 nm and emission 557–675 nm) were used. The DM 405/488/559/635 beam splitter and filters between the SDM490 and SDM560 channels were applied. The* z*-axis resolution was set at 2-μm steps and covered the entire specimen. FV viewer 4.2b software was used to acquire images of 1024 × 1024 pixel resolution. The resulting images were produced by merging the three channels (blue, green, and red). Images of the genital area were taken, and the samples were subsequently stored in Eppendorf tubes with Karnovsky fixative.

### Scanning electron microscopy

The male lice were removed from the Karnovsky fixative and washed in phosphate buffer for 3 h at 22 ± 1 °C. Samples were post-fixed with 2% osmium, washed with distilled water, then dehydrated with ethanol at increasing concentrations (30, 50, 70, 90, and 100%), and dried with CO_2_ using an Autosamdri-814 critical point dryer (Tousimis Research, Rockville, MD). The specimens were mounted on aluminum stubs using double-sided carbon tape. Next, the samples were coated with gold–palladium using a sputter coater (Polaron SC7640; Quorum Technologies, Laughton, East Sussex, UK) for 4 min. To improve conduction, a film of silver particles (OW52765459; Agar Scientific, Stansted, Essex, UK) was applied to the genitalia of nine specimens (two with partially extruded genitalia and seven with fully extruded genitalia). One specimen with fully extruded genitalia was not covered with the film. Images of the complete specimens and the specific structures of the genital area were taken. The samples were examined under a Hitachi S4800 scanning electron microscope (Hitachi High-Technologies) at an accelerating voltage of 10 kV and working distances of 8.7–10.2 mm. Images of 1280 × 960 pixel resolution were acquired.

## Results

### Specimens at rest versus specimens at the copulation stage

**Fig. 1 a, b:**
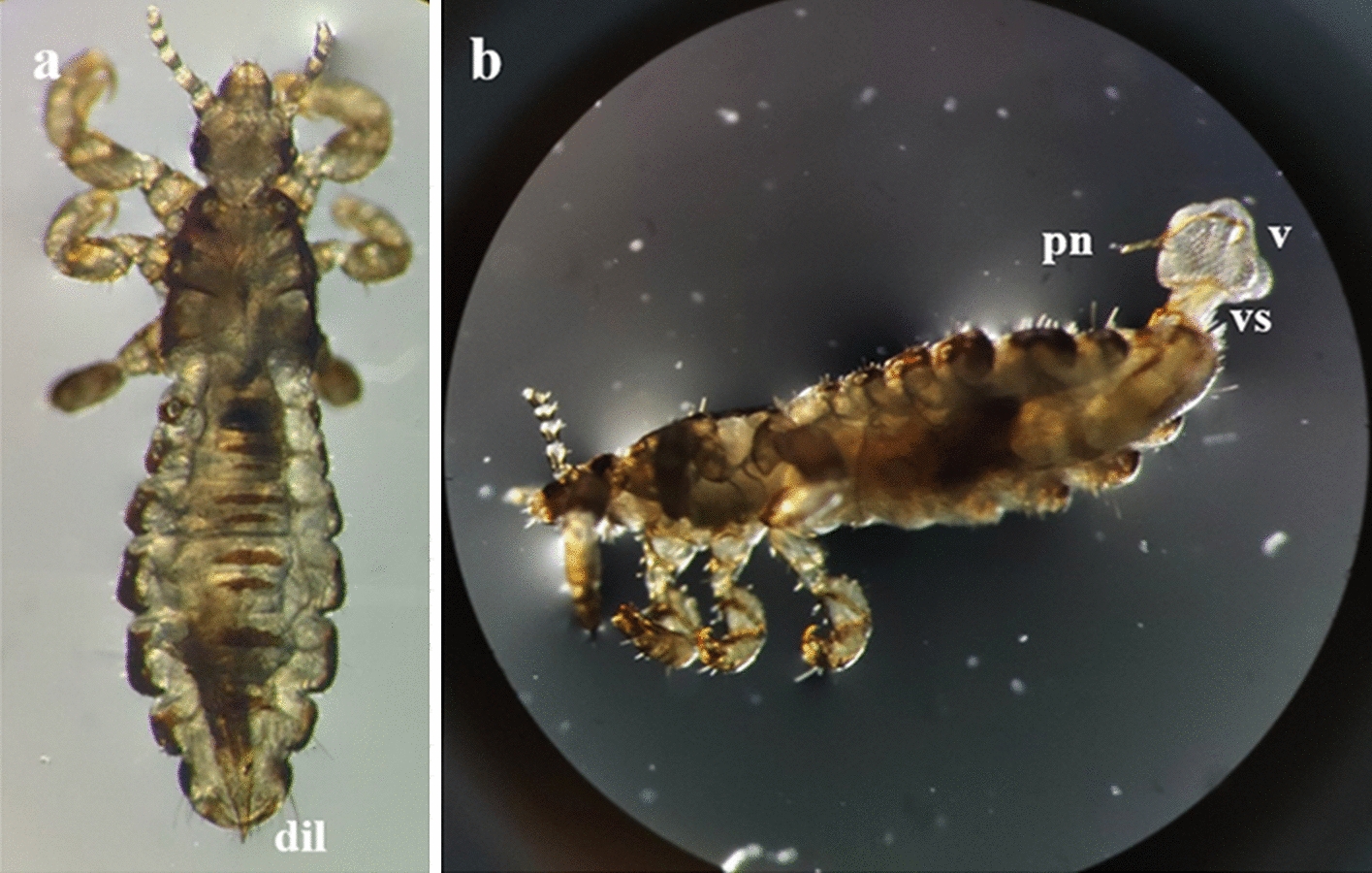
Male specimen of *Pediculus humanus capitis* under stereoscopic microscopy (SM). **a** Dorsal view of the distal end of the dilator (*dil*) at rest. **b** Male in copulation position; the vesica stem (*vs*), vesica (*v*), and penis (*pn*) can be observed. Abbreviations: dil, dilator; vs, vesica stem; v, vesica; pn, penis

Under SM, the genitalia of the specimens at rest were maintained within the genital chamber, with the apical part of the dilator being visible. In contrast, the specimens in the process of copulation had a visible dorsoventral inclination on abdominal segment VII and a translucid white pouch that protruded dorsally from abdominal segment IX. In this region, we identified the penis and two structures, namely the “vesica stem” and the “vesica” (Fig. 1a, b).

### Characteristics of the specimens at the copulation stage

**Fig. 2 a, b:**
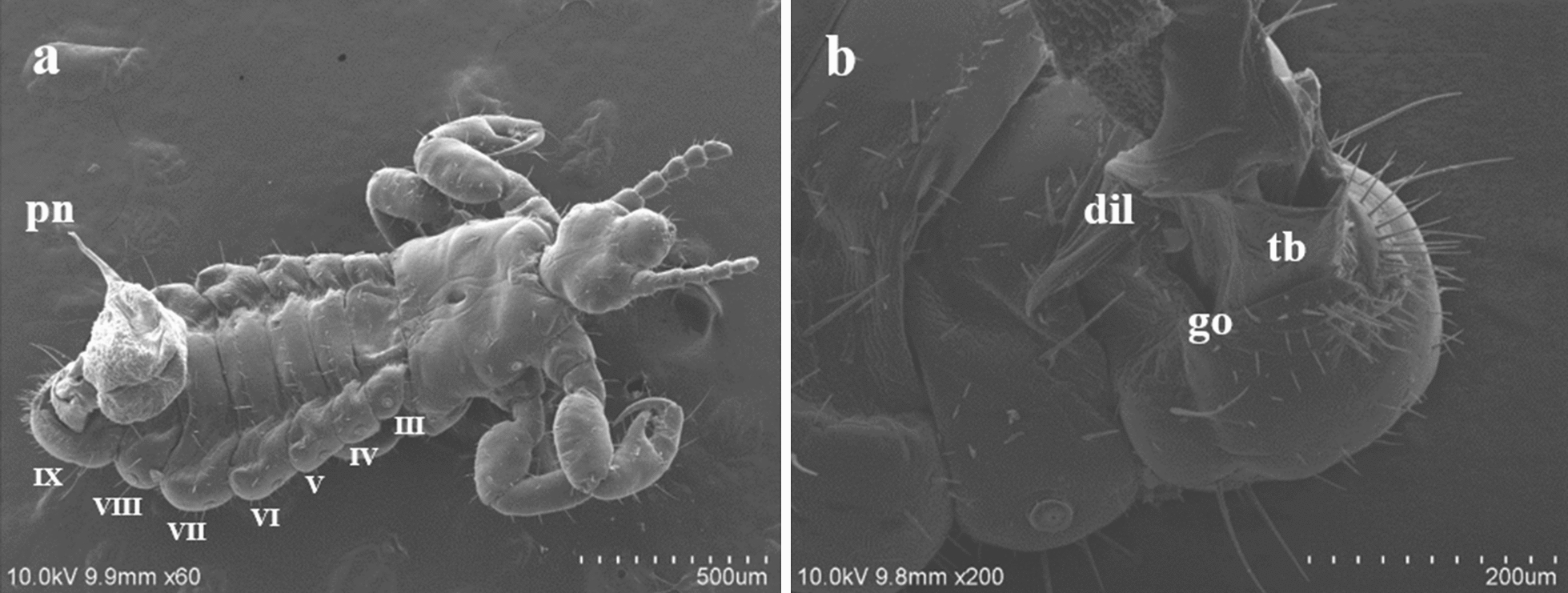
*Pediculus humanus capitis* under scanning electron microscopy (SEM). **a** Panoramic view of the extruded genitalia and abdominal segments III–IX. **b** Detail of segment IX showing the genital opening (*go*), the distal part of the tunica basalis (*tb*), and the dilator. For other abbreviations, see Fig. 1

**Fig. 3 a–d:**
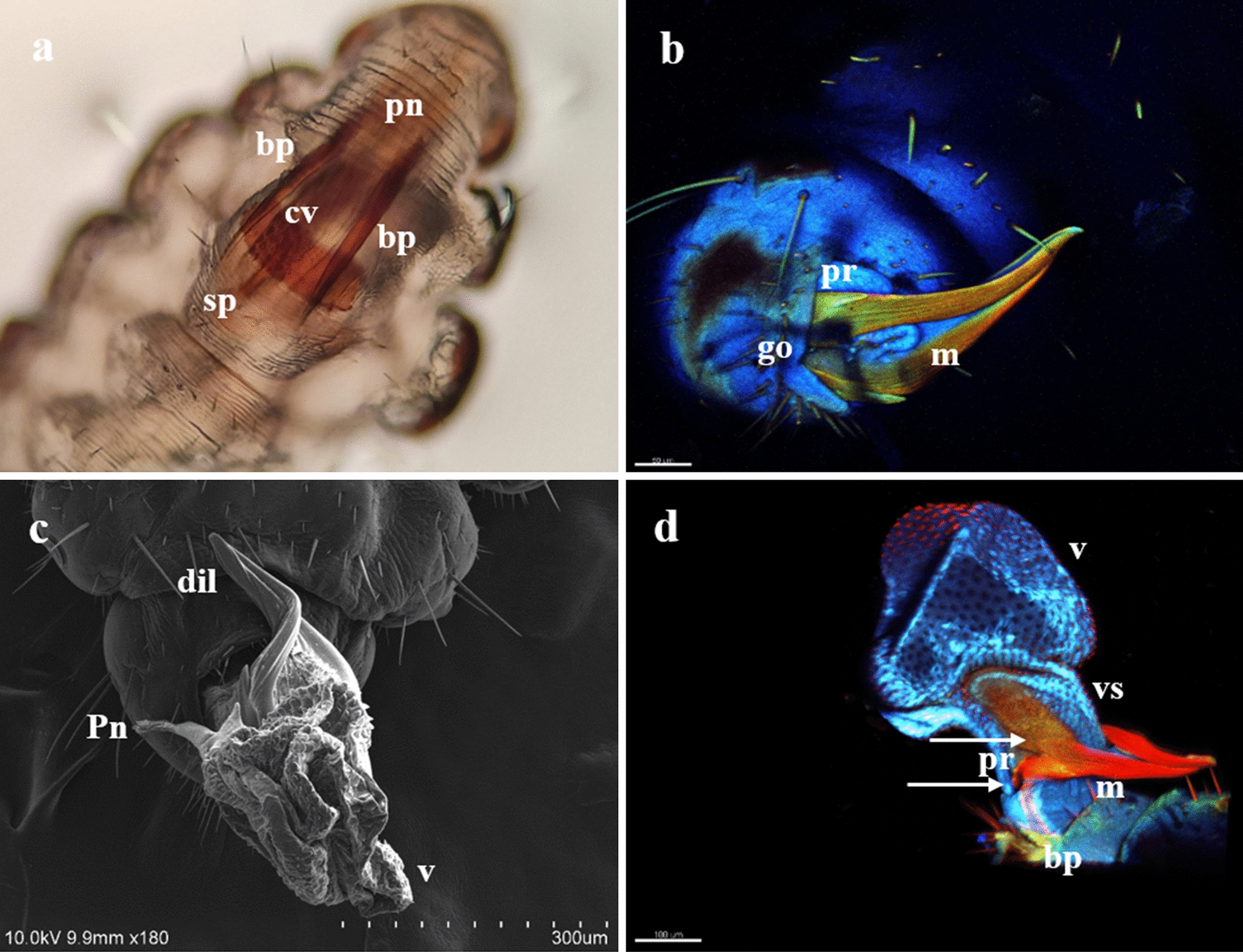
Structures of the male genitalia of *Pediculus humanus capitis* studied under SM, confocal laser scanning microscopy (CLSM), and SEM. **a** Ventral view of the genital chamber of a male at rest (SM microphotograph) showing the basal plate (*bp*), the contracted vesica (*cv*), the support of the penis (*sp*), and the penis, though the latter is less evident. **b** Male at the initial phase of copulation under CLSM. The relaxed genital opening, the mesomeres (*m*), and the parameres (*pr*) can be observed as a result of the position of the dilator.* Scale* 50 µm. **c** Detail of the position of the dilator during copulation, as seen under SEM. **d** Articulation of the parameres and the basal plate (*bottom arrow*), attachment of the mesomere to the vesica stem (*top arrow*), the vesica stem, and the vesica, as seen under CLSM.* Scale* 100 µm. For other abbreviations, see Figs. 1 and  2

At copulation, the male genitalia emerge through a horizontal opening, for which we propose the term “genital opening,” which is located dorsally to segment IX (Fig. 2a, b). This opening connects to the genital chamber, corresponding to abdominal segments VII–IX (Fig. 3a). The genital aperture had thick and smooth edges and was externally surrounded by setae. Short setae covered the anterior border, while those of varied sizes covered almost the entire posterior segment. Long setae were medial to the edge of the genital opening (Fig. 2b). In specimens at the initial stage of copulation, the dilator was anterodorsally inclined, which, combined with the relaxed genital aperture, allowed the genitalia to extrude (Fig. 3b).

### Structures that compose the genitalia

**Fig. 4 a:**
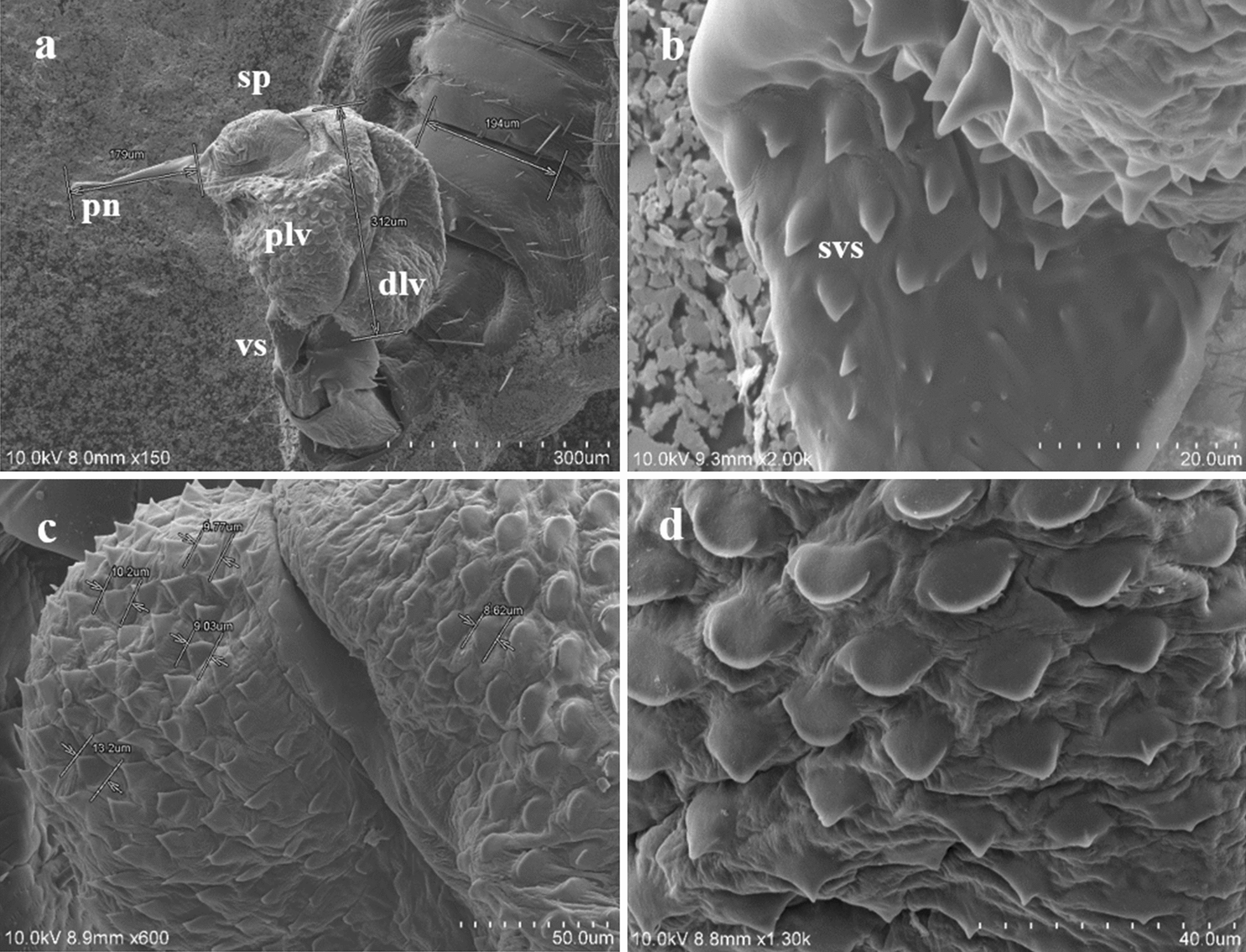
Detail of the structures of *Pediculus humanus capitis* under SEM. **a** General view of the genitalia: vesica stem, proximal lobe of the vesica (*plv*), distal lobe of the vesica (*dlv*), support of the penis, and penis. **b** Detail of the spines of the vesica stem (*svs*). **c, d** Details of the spines and scales of the vesica. For other abbreviations, see Figs. 1,  2 and 3

**Fig. 5 a–d:**
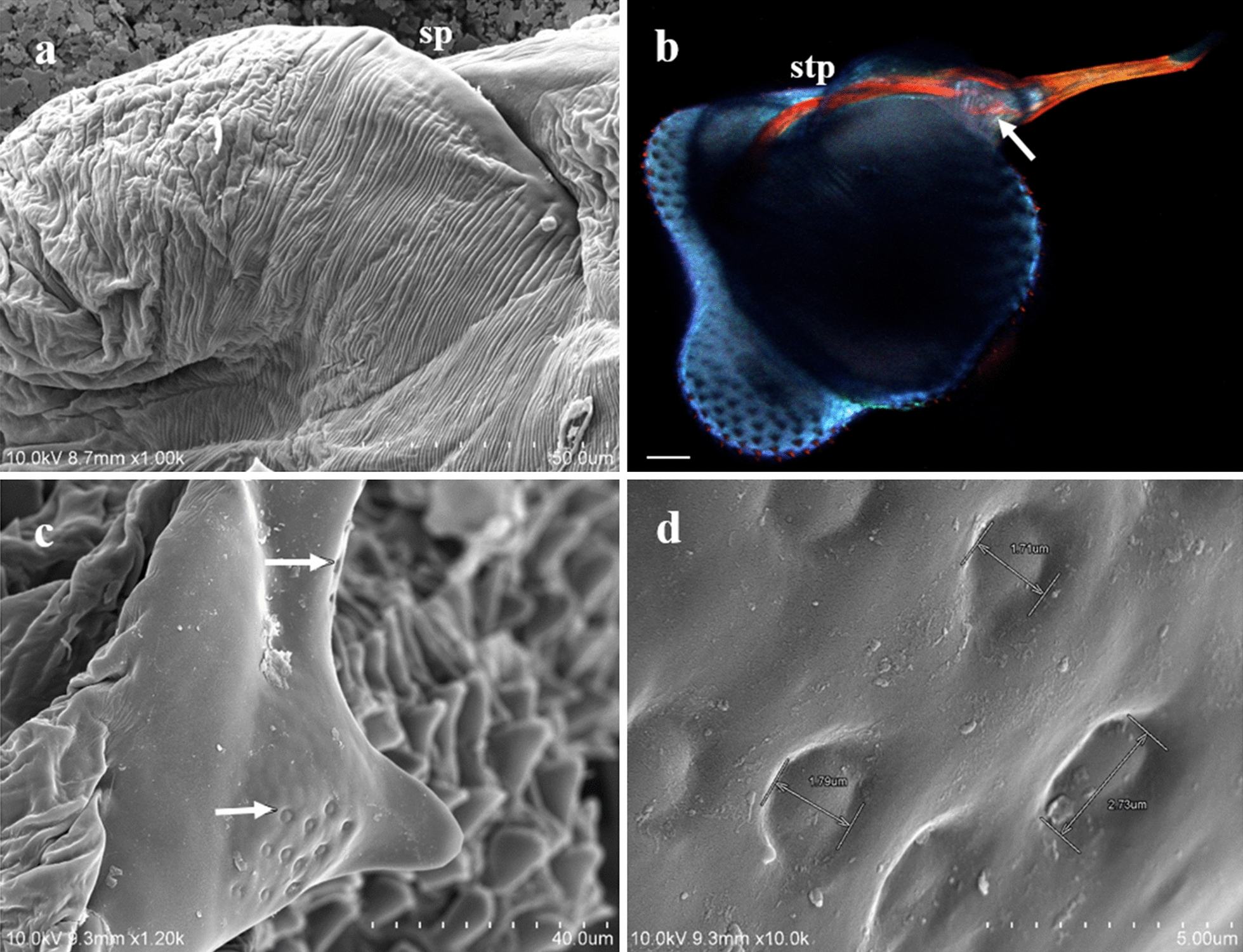
Anatomical details of the penis of *Pediculus humanus capitis*. **a** Support of the penis observed under SEM. **b** Supporting tendons of the penis (*stp*), seminal canal (*arrow*) observed under CLSM.* Scale* 50 µm. **c** Inverted crest of the penis with 11 depressions (*lower arrow*), some of the 14–16 orifices (*upper arrow*) observed under SEM. **d** Magnification of the depressions of the inverted crests observed under SEM. For other abbreviations, see Figs. 1,  2 and 3

**Fig. 6 a–d:**
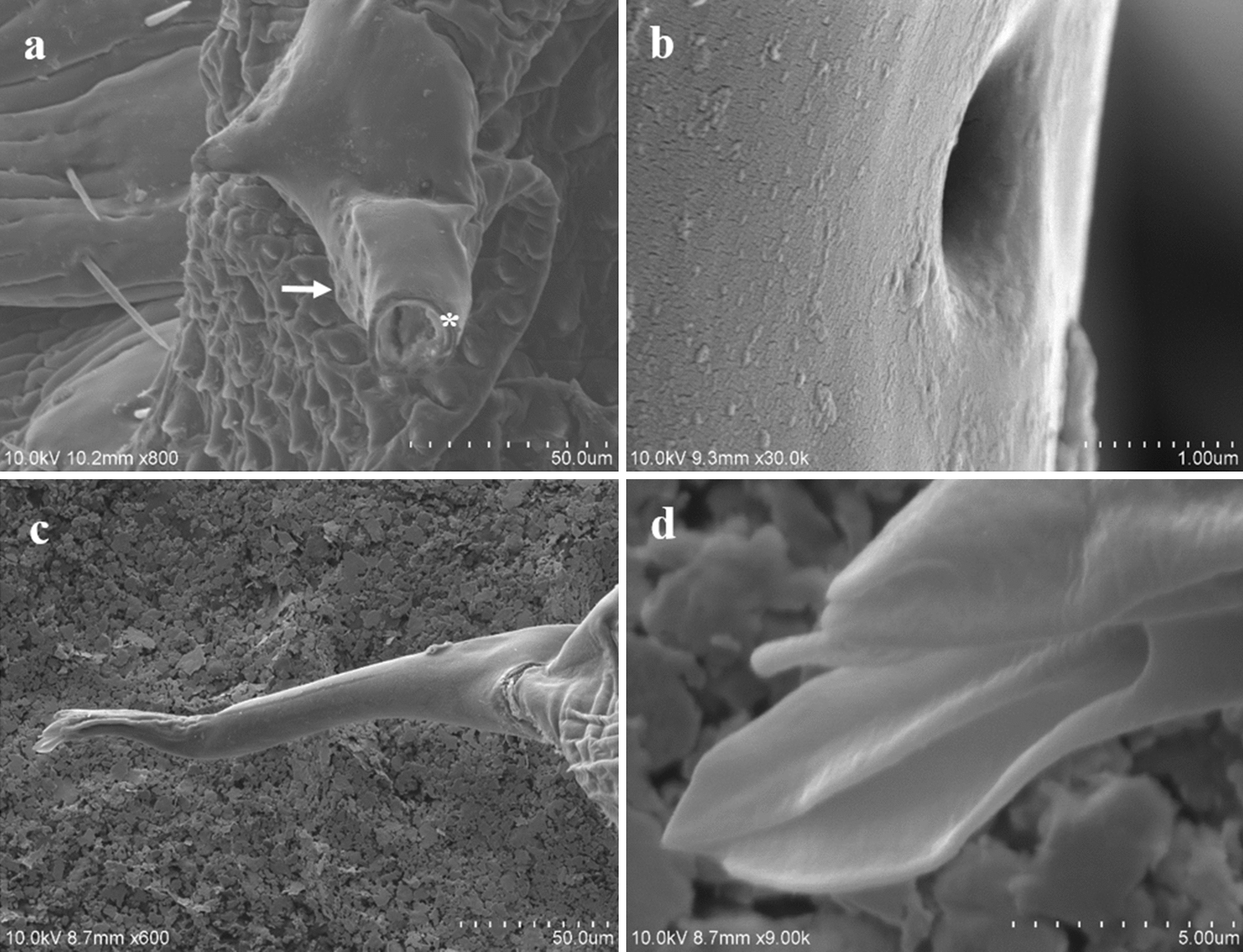
Morphological details of the penis of *Pediculus humanus capitis* under SEM. **a** The flat area with 14–16 orifices (*arrow*) at a cross-section of the penis (*asterisk*). **b** Magnification of the orifices. **c** Dorsal part of the penis. **d** Details of the foliaceous trifurcate tip

The male genitalia mainly consisted of the following structures:

1. The *tunica basalis*, a membrane with the aspect of a muscle that lines the genital chamber. We propose the term “tunica basalis” to describe this muscle-like membrane (Fig. 2b).
2. The* basal plate*, which is composed of a pair of rods positioned in a U shape at their proximal ends, and has thickenings laterally anchored to the tunica basalis (Fig. 3a).
3. The* dilator*, a structure formed by two arms, each of which is composed of a long mesomere partially fused with a short lateral paramere. The parameres are oriented to the posterior end of the basal plate. In the mesomeres, we observed attachments that are fixed laterally to the vesica stem. The arms of the dilator were found to fuse distally, forming a functional V-shaped structure with rounded ends. Posterior to the fusion point is a slightly left-oriented torsion that forms an angle with the penis. Ridges of variable size and depth are positioned lengthwise along the dilator (Fig. 3b, d).
4. The* vesica stem*, the structure that serves as a supportive base for the vesica and the penis. It is muscular and equipped with short, narrow spines (Figs. 3d, 4a, b).
5. The* vesica*, a structure composed of two lobes that are separated from each other by a fissure with heterogeneous borders with an average height of 312 μm. The proximal lobe (PLV) and the distal lobe (DLV) of the vesica are named according to their position relative to the penis (Fig. 4a). Two types of ornamentation were identified in the vesica: spine- and scale-like structures (average height 10.16 μm) with variable surface areas. The short and narrow spines are mainly distributed at the edges of the fissure that separates the two lobes of the vesica. Long and broad sharp-tipped or round-tipped scales are randomly distributed within the PLV and DLV. The round-tipped scales are predominantly located at the posterior medial region of the PLV, have a wavy base, and resemble a tongue (Fig. 4a, c, d). These ornamentations are partially visible when the vesica is contracted within the genital chamber (Fig. 3a).
6. The* support of the penis*, located on the dorsal part of the PLV. It is a hairpin-shaped structure free of spines or scales, lined with a contractile-like tissue, which serves as a supportive tissue for the penis (Figs. 4a, 5a). The presence of two interconnected thick ducts was verified by CLSM; these probably contribute to the stiffness of the penis. They run alongside the ejaculatory duct inside the PLV posterior wall and pass under the hairpin to reach the penis (Fig. 5b).
7. The* penis*, a smooth tubular structure, with the tip slightly curved upwards (average total length 179 μm). An extension was identified at its proximal end with the shape of an inverted crest with a gland-like portion. There, 11 shallow circular depressions with no apparent exit orifice were documented (Fig. 5c, d). Toward the apical end, a flat area with an average length of 2.07 μm was identified; it has 14–16 exit orifices of undetermined depth (Figs. 5c, 6a, b). The apical end of the penis has horizontal folds and terminates with a trifurcation that is leaf-like in shape. Cross-sections of the penis and the trifurcation revealed the presence of a hollow space, which possibly serves in the expulsion of the contents of the ejaculatory duct (Fig. 6a, b, d).

Our results show that the male genitalia of *P. humanus capitis* are complex but well-preserved structures since the morphological characteristics described here were consistently found in all the specimens studied.

## Discussion

In this article, we describe the structures of the male genitalia of *P. humanus capitis* and establish the morphological differences between the genitalia at rest (inside the genital chamber) and when they are extruded to prepare for copulation.

According to the microphotographs obtained with three different visualization methods, namely SM, CLSM, and SEM, the morphological evidence supports Nutall’s [10] observations for some of the studied structures. For this reason, we adopted his terminology, where appropriate, together with new terms that we have introduced to describe structures observed for the first time. The terms adopted from Nutall [10] include the dilator, vesica stem, vesica, penis support, and penis [10]. The terms we propose are the following: the tunica basalis; the proximal and distal lobes; the spines and scales of the vesica, which are microstructures; the spines of the vesica stem; the contractile lining that serves as a support for the penis; the inverted crest of the penis, which has 11 depressions; the flat area of the penis, which has 14–16 exit orifices; the foliaceous trifurcation at the apical end of the penis; and the hollow space that serves to expel the contents of the ejaculatory duct.

The term dilator is especially controversial due to the discrepancies that have been documented regarding its structure. It has been referred to by various terms, e.g., it was first called “dilator” [10], and later “pseudopenis” [12], “mesomere” [1], and “mesomeric arch” [9]. Nutall [10] opted for the term dilator since this structure serves to dilate the female’s vagina before copulation. He described its structure as composed of parameres distally fused with the lateral spurs. Despite the correct use of this term with respect to this structure’s function, anatomically it is not the most accurate. However, even though, from the results of the present study, we can confirm the observations of Retana-Salazar [9] concerning the existence of long mesomeres and short parameres, we still propose the use of the term dilator based on this structure’s function.

The basal plate is a structure that is widely conserved in the Psocodea. Since the position of this structure, as observed in our study, is homologous with that seen among different suborders of lice, we consider this term to be appropriate [1].

A 1998 study by Cicchino [14] that used SEM to examine the sucking louse of alpacas, *Microthoracius mazzai* (Phthiraptera: Anoplura), described a U- or V-shaped structure that the authors called a “protruding pseudopenis with a pointed appendix” and a second structure that they called a “rounded aedeagus” [14]. Judging from their morphology, the pseudopenis and aedeagus of *M. mazzai* may correspond, respectively, to the anterodorsally tilted dilator and the partially extruded internal genitalia observed here in *P. humanus capitis* (Fig. 3b).

Most studies on the genital structures of sucking and chewing lice provide illustrations of insects at rest [15, 16], which makes comparison and the establishment of homologies among the Phthiraptera difficult. Therefore, the use of new microscopic techniques applied at different stages of copulation is necessary to obtain a clearer view of the morphology of these structures and to avoid errors in the interpretation of their functions.

The combined use of advanced microscopic methods allowed us to visualize a multitude of penis structures in great detail. These structures and their characteristics were observed in all the specimens analyzed, showing that they are highly conserved. To our knowledge, some of these structures have not been reported previously for any suborder of lice.

The described genital structures seem to participate synergistically in the copulation of *P. humanus capitis*. It is possible that chemical signaling is involved, causing the dilator to respond and, at the same time, send signals to the structures within the genital chamber to prepare for extrusion. Supposing this signaling exists, the dilator, the vesica stem, and the vesica should present specialized sensory areas to receive this information. These sensory areas could be the spines and scales of the vesica stem and vesica. In addition, an internal pressure system and musculature that control the movement of the structures could also be involved.

The microscopic anatomy of the penis of *P. humanus capitis* has several interesting aspects; for example, the inverted crest that possesses an area with a glandular appearance, which is possibly related to the supply of nutrients and control of the pH balance of the louse’s semen. The flat area with the 14–16 exit holes probably plays a role in regulating the osmotic pressure exerted on the vesica which could ultimately lead to the expulsion of the semen from the penis.

Our results revealed the complexity of the male genitalia of *P. humanus capitis* when examined at different stages of copulation, and indicate that this should be taken into consideration in future investigations on the anatomy or the reproduction of similar species. In the present work, we provide the first description of the morphology of the male genitalia of *P. humanus capitis* using a new approach that combines different modern visualization techniques, and propose a change to the paradigm of the study of genitalia in lice. The application of state-of-the-art microscopic techniques to anatomical descriptions will undoubtedly impact future taxonomic and phylogenetic research on insects.

## Conclusions

The results of this study raise new questions regarding the function of some of the structures of the male genitalia of *P. humanus capitis* and their underlying mechanisms. Future investigations should aim at unraveling whether structures such as the dilator and the vesica spines or scales have sensory sensitivity to chemical signals that are involved in sexual activity, which might imply their participation in the processes of mating and associated rituals. Other structures of interest for further study are the inverted crest of the penis and the orifices of the flat area, which, due to their location, are possibly related to the proportions of nutrients and the pH balance of the louse’s semen. Detailed descriptions of the physiological functions and signaling mechanisms of each structure would enable the comprehensive description of the reproductive system of the male of *P. humanus capitis*.

## Data Availability

Data supporting the conclusions of this article are included within the article. The raw datasets used and analyzed during the present study are available from the corresponding author upon reasonable request.

## Abbreviations

SM: Stereoscopic microscopy
CLSM: Confocal laser scanning microscopy
SEM: Scanning electron microscopy
